# Mathematical modelling of reoviruses in cancer cell cultures

**DOI:** 10.1371/journal.pone.0318078

**Published:** 2025-04-28

**Authors:** Arwa Abdulla Baabdulla, Francisca Cristi, Maya Shmulevitz, Thomas Hillen

**Affiliations:** 1 Department of Mathematical Sciences, United Arab Emirates University, Al Ain, United Arab Emirates; 2 Department of Medical Microbiology and Immunology, University of Alberta, Edmonton, Alberta, Canada; 3 Li Ka Shing Institute of Virology, University of Alberta, Edmonton, Alberta, Canada; 4 Cancer Research Institute of Northern Alberta, University of Alberta, Edmonton, Alberta, Canada; 5 Department of Mathematical and Statistical Sciences, University of Alberta, Edmonton, Alberta, Canada; Keele University School of Life Sciences, UNITED KINGDOM OF GREAT BRITAIN AND NORTHERN IRELAND

## Abstract

Oncolytic virotherapy has emerged as a potential cancer therapy, utilizing viruses to selectively target and replicate within cancer cells while preserving normal cells. In this paper, we investigate the oncolytic potential of unmodified reovirus T3wt relative to a mutated variant SV5. In animal cancer cell monolayer experiments it was found that SV5 was more oncolytic relative to T3wt. SV5 forms larger sized plaques on cancer cell monolayers and spreads to farther distances from the initial site of infection as compared to T3wt. Paradoxically, SV5 attaches to cancer cells less efficiently than T3wt, which lead us to hypothesize that there might be an optimal binding affinity with maximal oncolytic activity. To understand the relationship between the binding process and virus spread for T3wt and SV5, we employ mathematical modelling. A reaction-diffusion model is applied, which is fit to the available data and then validated on data that were not used for the fit. Analysis of our model shows that there is an optimal binding rate that leads to maximum viral infection of the cancer monolayer, and we estimate this value for T3wt and SV5. Moreover, we find that the viral burst size is an important parameter for viral spread, and that a combination of efficient binding and large burst sizes is a promising direction to further develop anti-cancer viruses.

## 1 Introduction

Oncolytic virotherapy has emerged as a compelling strategy for addressing the challenges of cancer treatment. By leveraging the ability of oncolytic viruses to selectively target and replicate within cancer cells, this approach aims to achieve tumor cell lysis without causing harm to healthy cells [1–3]. The consequential activation of the host immune system, promoting an anti-cancer immune response, further adds to the potential therapeutic benefits [4].

Various oncolytic viruses, including adenoviruses [5–9], herpes simplex virus [10–13], vaccinia viruses [14–16], measles virus [17,18], vesicular stomatitis virus (VSV) [19,20] and reovirus [21–26] have been investigated for their suitability in oncolytic virotherapy. Viruses are genetically modified to enhance their oncolytic potential and ensure selective targeting of cancer cells. Preclinical studies utilizing in vitro and in vivo models have been conducted to evaluate the safety and efficacy of these modified viruses [27,28].

In this research, we focus on the oncolytic potential of reovirus [29–33]; a double-stranded RNA nonpathogenic virus with natural tropism to the enteric tract of mammals. The unmodified laboratory strain of reovirus serotype 3/T3DPL (T3wt) has demonstrated natural capabilities for infecting and lysing tumors under both in vitro and in vivo conditions [21,25,26,34,35]. T3wt is currently undergoing evaluation in over 30 clinical trials targeting various cancer types, including metastatic breast cancer [35,36], prostate cancer [37,38], and colorectal cancer [21,39]. Additionally, it has progressed to phase III clinical trials as a potential therapeutic intervention for breast cancer [36,40].

While T3wt is consistently well-tolerated [5,6,27,41,42], the majority of patients do not respond to reovirus therapy, and overall responses remain underwhelming [2]. A recent summary of oncolytic viral clinical trials as a whole similarly highlight that only  ∼  21% of patients show some response to oncolytic viruses [7]. Mutants of T3wt have therefore been selected to enhance oncolytic activity on cancer cells in vitro and improve tumor regression and survival in animal tumor models in vivo [25,29–32,40]. One such mutant, SV5, demonstrated significant improvement of oncolysis in vivo that correlated with key parameters in cell culture including binding percentage, the distance of virus spread from primary sites of infection, and plaque sizes in cell monolayer experiments [25,26]. The outcomes of these assessments underscore the pivotal importance of enhancing the distance that oncolytic viruses travel before re-infection as a critical mechanism for optimizing therapeutic interventions in the context of oncolytic virotherapy. Intriguingly, the experimental results reveal a discernible connection between the binding rate and the distance a viral infection spreads over the cell monolayer, suggesting that a reduction in the binding rate can lead to more extensive and distal viral spread, characterized by larger plaque sizes. This observation highlights a distinct advantage that the mutated SV5 virus possesses over the wild-type T3wt, despite similarities in viral production and cell death. These findings motivate the mathematical question addressed in this study, which is, what are the optimal values of virus binding rates that retain sufficient cell attachment to permit efficient infection of cells but also allow further distance of virus spread before re-infection to produce larger areas of virus dissemination?

The phenomenon of viruses exhibiting reduced binding to host cells has been extensively documented in the scientific literature [42–49]. Notably, reovirus variants with diminished affinity for sialic acid have been identified in both murine and human species. A sialic-acid-binding-deficient reovirus variant exhibited heightened infectivity when compared to the wild-type reovirus in polarized epithelial cells from apical or basolateral orientations [43]. In the context of rotavirus mutants incapable of binding to sialic acid, although these mutants displayed slower replication and lower titres in mouse cancer cell lines MA104, they paradoxically exhibited increased pathogenicity in mice [44]. This underscores the nuanced relationship between viral binding capabilities and infection outcomes under specific conditions.

Expanding beyond reoviruses and rotaviruses, other viruses have demonstrated the capacity to spread more extensively in monolayer cell cultures without a concomitant increase in replication. For instance, vaccinia virus-infected cells repel superinfecting virions, resulting in enhanced viral spread [45]. Additionally, reduced adsorption rates to host bacteria have been linked to increased plaque size in phages [42]. Moreover, various virus variants of polyomavirus, parvovirus, and Sindbis virus, characterized by deficiencies in binding, have been shown to generate larger plaques in vitro [46–49]. Importantly, these variants exhibited higher pathogenicity and increased spread in vivo.

Building on experimental data from [26] we address key questions through mathematical modeling: How does the viral spread distance correlate with the binding rate? What is the dependence of the viral spread rate on the binding rate? How does the reduction in binding rate impact plaque sizes in vitro experiments? Is there an optimal binding rate, which maximizes viral infection of the cell monolayer? Mathematical models in forms of reaction-diffusion equations for viral plaque size experiments have been used before in [50–52] and our model follows those principles.

To answer the above questions, we use three mathematical models that capture different aspects of viral dynamics. Model 1 focuses on a small-time scale (less than 16 hours), enabling observation of viral spread distances without considering cell death and viral replication. Model 2 extends the analysis to a longer time scale (about 5 days), incorporating viral spread, cell death, and viral replication. Model 3 then includes the cancer cells explicitly, which allows us to compare plaque sizes of different experiments. The results of our models exhibit excellent concordance with the observed experimental phenomena, providing valuable insights into the dynamics of reovirus-mediated oncolytic therapy. Based on the modelling we are then able to compute the optimal binding rate that leads to the largest plaque size.

### 1.1 Mathematical modelling of oncolytic virotherapy

Mathematical modeling plays a critical role in advancing oncolytic virotherapy research by elucidating key parameters, generating novel testable hypotheses, predicting therapeutic outcomes in silico, and optimizing combination treatments. The majority of existing models have focused on the temporal dynamics of tumor-virus and tumor-virus-immune interactions, primarily due to the availability of temporal data. These models predominantly employ ordinary differential equations (ODEs) [53–61], while others are formulated using delay differential equations [62–66].

Wodarz et al. [67] was the first to develop an agent-based model to explore the viral propagation patterns in tumor cell populations, specifically using adenoviruses in a two-dimensional setup of human embryonic kidney cells. Their model considered a spatially constrained environment, assuming free viruses were in a quasi-steady state. The experimental results showed three spatial patterns: "hollow ring structure," "filled ring structure," and "dispersed patterns." The simulations indicated that the hollow ring structure was optimal for treatment, as it often led to the extinction or low persistence of target cells. Building upon this work, Rioja et al. [63] derived a continuum model with radial symmetry, where the viral dynamics expressed explicitly. The corresponding kinetic ODE system derived from Rioja et al.’s model was subsequently examined in [59]. A three-dimensional spherical symmetric model was explored by Pooladvand et al. [68] to elucidate the dynamics of adenovirus in a spherical glioblastoma. Their focus was on the impact of the infectivity parameter on treatment outcomes, with virus injection occurring at the tumor’s center. Results indicated that increased infectivity did not lead to complete tumor eradication, aligning with experimental findings that suggest monotherapy with virotherapy is often insufficient. The model of Pooladvand was further analysed in Baabdulla and Hillen [69], where the tumor control probability was used to identify the hollow-ring pattern as the most beneficial state. In addition, chaotic long-time dynamics were found in 2-dimensional simulations.

Bhatt et al. [70] investigated factors behind virotherapy failures and ways to improve treatment efficacy, emphasizing tumor cell sensitivity to viral infection. Using an immersed boundary method and spatial interaction models, they identified three main reasons for treatment failures: high mortality of infected cells leading to rapid viral clearance, emergence of virus-resistant cancer cells, and insufficient viral spread rates. On the other hand, Morselli et al. [71] introduced a stochastic agent-based model to study how spatial constraints and the tumor microenvironment affect viral dissemination within solid tumors. Their two-dimensional simulations echoed the spatial patterns identified by Wodarz et al. [67] and Kim et al. [72].

To elucidate the immune system’s role in virotherapy, several models have been developed in the literature [55,56,61,73] and references therein. A seminal study by Storey et al. [61] formulated an ODE framework to explore the contributions of innate and adaptive immune responses to virotherapy in glioblastoma multiforme treated with Herpes Simplex Virus. Their model delineated two roles for the innate immune response: acting as an immediate responder against virus-infected cells and stimulating the adaptive immune response mediated by T-cells. They found that under virotherapy alone, the innate immune system’s virus clearance capability predominates, leading to rapid viral elimination and minimal impact from the adaptive response. To enhance the adaptive immune response, they examined a combination of virotherapy and immunotherapy through PD-1/PD-L1 inhibition, which is critical in preventing cancer cells from evading T-cell activity. Their findings demonstrated significant benefits from this combinatorial approach. In a subsequent study, Storey and Jackson [73] expanded their model to a spatially explicit agent-based framework, highlighting the necessity of administering viral doses at sites of maximum tumor density rather than at the tumor’s center for effective treatment outcomes.

Our modelling is based on the models discussed above. The emphasis here is not on new model development, rather, the fitting of the models to experimental data on reovirus, explanations of the observed properties, and subsequent suggestions of further improvements and further experiments.

### 1.2 Summary of experimental observations of [26]

Our mathematical models will be fitted and validated from the data of [26], where we have access to the original data set. In [26], characteristics of T3wt and SV5 are empirically determined on monolayers of TUBO cells (spontaneously derived HER2/neu positive murine breast cancer cells) and L929 cells (tumorigenic mouse fibroblasts). Monolayers of TUBO and L929 cells are exposed to T3wt or SV5 to measure cell attachment, virus replication, cell killing, and the size of plaques produced over several rounds of infection and re-infection. Based on the experiments reported in [26,30], reovirus typically bind cells within an hour, enter in 3-4 hours, replicate exponentially until 15-18 hours, and newly-made viruses become released from cells at 18-20 hours post-infection (hpi). Accordingly, short-term infections of 1 hour are used to monitor binding. Long-term infections of up to 5 days are used to monitor plaque size. In [25] they find that among a variety of reovirus mutations, the variant SV5 (supervirus 5), which has five mutations in the virus genome, leads to the largest plaque sizes. Relative to T3wt, SV5 displays similar kinetics of replication in an infected cell, cell death of the infected cell, burst size (the titer of virus released from infected cells), and diffusion. However, SV5 bindings less efficiently to cells, and also produces significantly larger plaques over several rounds of infection. While our mathematical modelling will use the empirical data derived on cancer cell cultures, it might be of interest to the reader that SV5 also significantly improves tumor regression and mouse survival in the more-complex mouse models of TUBO-derived tumors.

### 1.3 Outline

The paper introduces a multi-scale mathematical modeling approach to investigate the dynamics of viral spread in cell culture experiments. The study begins with a short time scale model in Sect 2, utilizing data from [26] to estimate the binding rate $\gamma_{b}$ and viral diffusion coefficient $D_{\text{V}}$. Sect 3 extends the model to longer time scales, incorporating events such as cell death and viral production. The speed of the spread of viral infection over the monolayer is computed via a travelling wave analysis, revealing the relationship between the binding rate $\gamma_{b}$ and the viral spread speed $c^{*}$ in Sect 3.6. Model validation is presented in Sect 3.7, where additional experimental data is considered. In Sect 3.8 and Sect 4, we present the main result on the optimal choice of the binding rate $\gamma_{b}$. The paper concludes in Sect 5, contextualizing the results within the broader scope of mathematical modeling, and cancer and viral infection research.

## 2 Model 1: Short time scale

**Fig 1:**
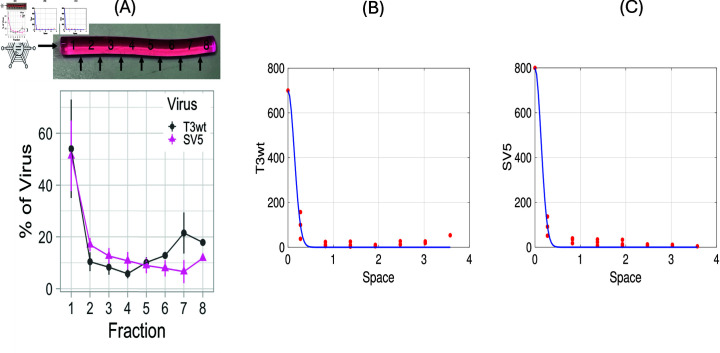
The diffusion coefficient estimation for T3wt and SV5 particles. (A): Percent of total T3wt and SV5 virus particles, where the syringe of length 4.4 cm is divided into 8 fractions, measured following 120 hours of incubation [26], picture taken from Fig 7A in [26]. (B): Plot of the original percentage data from [26] for T3wt and our fit as solid line. (C): Plot of the original percentage data from [26] for SV5 and our fit as solid line.

First, we start by answering the following question: How far does the virus spread depending on the binding rate during a time that is short enough to exclude effects of long-term cell death and viral replication? To answer this question, we use two sets of data from [26]. In short-time experiments, the percentage of virus binding to L929 cells after 1 hour was measured (see Table 1). These data will be used to estimate the binding rate $\gamma_{b}$. Second, to estimate viral diffusion in the extracellular medium, the viral load was measured after inoculation in a cell free medium (see Fig 1).

**Table 1:** The percentage of binding for each virus type at 1 hour for three data sets each; taken from the original data that were used for Fig 6A in [26]. The estimated data range is computed.

Virus Type	*%* of Binding	Mean ± Error	Half-life Time $t_{1∕2}$ (Conf. Int.)
T3wt	80.64	61.63 ± 10.14	0.722 hours (0.619,0.866)
T3wt	58.25		
T3wt	45.99		
SV5	24.25	24.18 ± 5.75	2.476 hours (1.873, 3.648)
SV5	34.10		
SV5	14.19		

On the short time scale of 1-16h, we consider only two processes, which is binding of the virus to the cells and diffusion of virus particles in the cell medium. Hence our model has the form


$$\begin{array}{ccc} \frac{\partial V}{\partial t}=D_{\text{V}}\Delta V-\gamma_{b}V, & & \end{array}$$ (1)

with the initial condition


$$\begin{array}{ccc} V(x,y,0)=V_{0}\delta_{0}(x,y). & & \end{array}$$ (2)

Here *V* ( *x* , *t* )  denotes the titre of virus particles, $D_{\text{V}}$ is the constant diffusion coefficient, $\gamma_{b}$ is the constant binding rate and $V_{0}$ is the amount of virus particles at the start of the experiment *t* = 0. The symbol *Δ* denotes the Laplace operator, which is the sum of all second order partial derivatives, and $\delta_{0}(x,y)$ denotes the delta distribution, which indicates the point of viral injection at the beginning of the experiment. We use model (1) to estimate the binding rate $\gamma_{b}$ and the diffusion coefficient $D_{\text{V}}$.

### 2.1 Binding rate estimation

To measure the binding rate $\gamma_{b}$, we use the data in [26], where three experiments for each reovirus type were completed to estimate the efficacy of reovirus attachments to tumor cells. L929 cells were exposed to equivalent virus particle doses and incubated at $4^{º}C$ for 1 hour to enable virus attachment without entry into the cells (i.e entry requires temperatures above $19^{º}C$). The unbound virus particles were removed by washing the cells extensively before harvesting the post-binding lysates. Finally, Western blot analysis was used to calculate the percentage of cell-bound virus particles based on virus protein levels in the lysates versus the input. The results show that on average 62*%* of T3wt virus were bound to L929 cells, compared to on average 24*%* of SV5 virus particles bound to cells (Table 1).

The binding process can be easily described by a linear binding law


$$\frac{d}{dt}V(t)=-\gamma_{b}V(t),\;V(0)=V_{0}.$$

To estimate the binding rate $\gamma_{b}$, we assume that the data in Table 1 are normally distributed and apply the likelihood method with the least square error (LSE) [74]. We denote the measured *%*-values of viruses binding as $y_{i},$ for i=1,2,3, as there are three independent data points for each virus. The solution to the above equation is an exponential, which we can use to compute the number of unbound virus particles at time *t* as


$$\begin{array}{ccc} V(t)=V_{0}e^{-\gamma_{b}t}. & & \end{array}$$ (3)

Then, the percentage of bound virus particles after *t* = 1 hour is


$$y_{i}=1-e^{-\gamma_{b}}.$$

Therefore, $\gamma_{b}=-\text{ln}(1-y_{i}).$ As there are several measurements for $y_{i}$, an average is necessary, which is generated using the maximum likelihood estimator.


$$\begin{array}{ccc} \tilde{\gamma_{b}}=\ln\left(\frac{1}{k}\overset{k}{\underset{i=1}{\sum }}(1-y_{i})\right). & & \end{array}$$ (4)

The corresponding confidence interval is then computed (see [74]). This estimation of the binding rate finds for the wild type (T3wt) $\gamma_{b\text{T3wt}}=0.96\;\pm \;0.16$ per hour, while for SV5 it is $\gamma_{b\text{SV5}}=0.28\pm 0.09$ per hour.

These values can be related to the half-life of the virus population by


t1∕2=ln ⁡ 2γb.

The T3wt virus population has a shorter time for binding with $t_{1∕2}=0.722$ hours compared to the SV5 virus population with $t_{1∕2}=2.476$ hours. Our results are consistent with the overserved measurements, as the data results indicated that T3wt virus particles have a higher percentage of binding with more than 50*%* of them binding compared to SV5 in one hour.

### 2.2 Diffusion coefficient estimation

The spatial diffusion of virus inside the culture medium without host cells was measured using the barrel of 1 mL syringes filled with semi-solid 0.5 % agar medium. The virus was introduced at the top of the medium and allowed to diffuse over time. The medium then removed from syringes and divided spatially into equal fractions (see Fig 1 A). Each fraction corresponds to 100 *μ*L .  The percentage of viral load that diffused into each fraction was measured at time *t* = 120 hours. The spatial extent of each fraction is approximately 0.55 cm as we can see in Fig 1 A. To estimate the diffusion coefficient $D_{\text{V}}$ at t=120 hour, we fit the data in Matlab by applying the Gaussian distribution formula for a diffusion process.


$$\begin{array}{ccc} V(x)=\frac{V_{0}}{\sqrt{4\pi D_{\text{V}}t}}e^{\frac{-x^{2}}{4D_{\text{V}}t}}. & & \end{array}$$ (5)

The parameters $V_{0}$ and $D_{\text{V}}$ are then estimated, where $V_{0}$ is the number of viruses particles at t=0 which was not measured in the experiments. In Fig 1 we show the Fig 7A from [26] in (A), and replot the original data with our fits in (B) and (C). We find that there were no significant differences in the diffusion coefficients between T3wt and SV5 viruses. The best fit estimated diffusion coefficient in both cases is $D_{\text{V}}=0.01\;\pm \;0.0015\;{\text{mm}}^{2}\;\text{per}\;\text{hour}$ (equivalent $D_{\text{V}}=0.0001\;\pm \;0.000015\;{\text{cm}}^{2}$ per hour) (Fig 1B and 1C). The estimated values for $V_{0}$ differ slightly, $V_{0,T3wt}=243$ and $V_{0,SV5}=265$. Note that $V_{0}$ is not the value of *V* at location *x* = 0. Rather, $V_{0}$ denotes the initial amount of viral inoculation that occured at the initial time at *x* = 0. As such, the integrals under the curves of Fig 1 give the corresponding $V_{0}$ values.

Another way to evaluate the diffusion coefficient $D_{\text{V}}$ of small particles in a medium is by applying the Stokes-Einstein equation for the diffusion coefficient $D_{\text{V}}$ of a spherical particle of radius *r* in a fluid of dynamic viscosity *η* at absolute temperature *T* [75]. We do not have any direct information from the data to estimate the value of the viscosity of the 0.5 % agar in Minimum Essential Media (MEM). Therefore, we use the viscosity of water which is also similar to the viscosity of Dulbecco’s Modified Eagle Medium (DMEM) (10 % FBS where FBS refers to a Fetal Bovine Serum) [41]. Thus, in our case the Stokes-Einstein relation gives


$$D_{\text{V}}=\frac{k_{B}T}{6\pi \eta r}=0.02\;mm^{2}\;\text{per hour},$$

where $k_{B}$ is Boltzmann’s constant, *r* = 35nm [76] is a typical virus radius, and *η* = 0 . 001pa . s is the viscosity of water at $T=21^{º}C.$ The values of diffusion coefficient in our estimation and Stokes-Einstein are very close. Furthermore, additional studies [63], [68] use a similar diffusion coefficient of $D_{\text{Rioja}}=0.014\;mm^{2}$ per hour and $D_{\text{P}}=0.01\;mm^{2}$ per hour for cancer viral therapy, which are similar to our value $D_{\text{V}}=0.01\;mm^{2}$ per hour as we can see in Table 2.

**Table 2:** Comparison between the value of the viral diffusion coefficient estimated in our model versus the Stokes-Einstein relation, by the Rioja et al. model [63], and the Poolandvand et al. model [68].

$D_{\text{ V}}\;({\text{mm}}^{2}\;\text{per}\;\text{hour}$)	
Our model fit estimation	0 . 01 ± 0 . 0015
Stokes-Einstein estimation [75]	0.02
Rioja et al. [63] estimation	0.014
Poolandvand et al. [68] estimation	0.01

### 2.3 Result: Prediction of the spread radius for short times

Based on the above modelling and parameters we can estimate the distance a viral inoculation should spread on a short time scale. We assume the inoculation is somewhere in the centre of the monolayer such that viral spread is essentially radially symmetric. We consider the spread radius as the maximum distance from the inoculation after which no virus particles are detectable. The critical detection threshold for virus titer is denoted as $V_{\text{min}}$. Hence we ask the question, for which radius *r* is the virus titer equal to $V_{\text{min}}$?

For this we solve Eqs (1)–(2) with a little trick by setting $\phi =Ve^{\gamma_{b}t}$. Then


$$\phi_{t}=V_{t}e^{\gamma_{b}t}+\gamma_{b}Ve^{\gamma_{b}t}=e^{\gamma_{b}t}\left(V_{t}+\gamma_{b}V\right)=D_{\text{V}}e^{\gamma_{b}t}\Delta V=D_{\text{ V}}\Delta \phi$$

Hence *ϕ* satisfies a linear heat equation, which we can solve explicitly using the fundamental solution in 2-D [74].


$$\begin{array}{lll} \phi (x,y,t)=\frac{V_{0}}{4\pi D_{\text{V}}t}e^{\frac{-x^{2}-y^{2}}{4D_{\text{V}}t}}. & \; & \end{array}$$

Using $\phi =Ve^{\gamma_{b}t},$ we obtain


$$\begin{array}{ccc} V(x,y,t)=e^{-\gamma_{b}t}\frac{V_{0}}{4\pi D_{\text{V}}t}e^{\frac{-x^{2}-y^{2}}{4D_{\text{V}}t}}. & & \end{array}$$ (6)

To compute the spread radius $r=\sqrt{x^{2}+y^{2}}$, we assume that below a level of $V_{\text{min}}$ no virus can be measured. Hence at the spread radius *r* we have


$$V_{\text{min}}=e^{-\gamma_{b}t}\frac{V_{0}}{4\pi D_{\text{V}}t}e^{\frac{-r^{2}}{4D_{V}t}}.$$

We solve this equation for *r* and obtain


$$\begin{array}{ccc} r=\sqrt{4D_{\text{V}}t\left(\ln\left[\frac{V_{0}}{4\pi D_{\text{V}}V_{\text{min}}t}\right]-\gamma_{b}t\right)}. & & \end{array}$$ (7)

**Fig 2:**
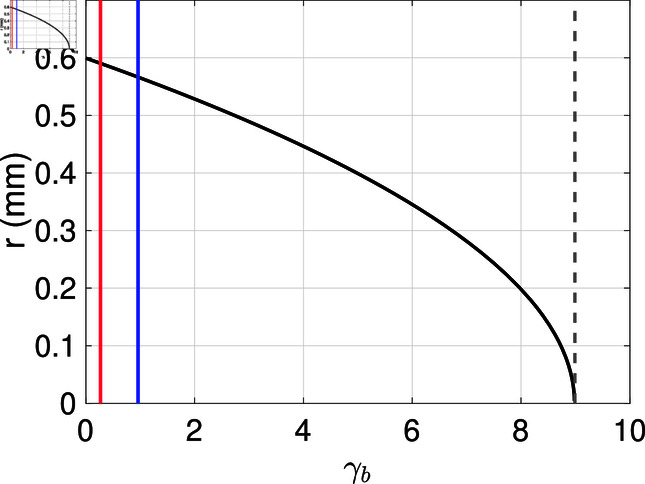
The plaque radius values as function of the binding rate $\gamma_{b}\;(\text{per}\;\text{hour})$ with $D_{\text{V}}=0.01\;{\text{mm}}^{2}\;\text{per}\;\text{hour},$ and $\frac{V_{0}}{V_{\text{min}}}=1000$ virus at t=1 hour. The blue line represents the value of $\gamma_{b\text{T3wt}}=0.96,$ while the red line represents the value of $\gamma_{b\text{SV5}}=0.28.$

Fixing the time *t*, the diffusion coefficient $D_{\text{V}}$, the number of virus particles at t = 0 i.e $V_{0},$ and the threshold $V_{\text{min}},$ we have the spread radius *r* as a function of the binding rate $\gamma_{b}$. We show this dependence in Fig 2. In Fig 2 we also show the estimated binding rate for the wild type virus in blue ($\gamma_{b\text{T3wt}}=0.96\;\text{per}\;\text{hour}$) and for SV5 in red ($\gamma_{b\text{SV5}}=0.28\;\text{per}\;\text{hour}$). Furthermore, we see that the spread radius declines as the binding rate increases. For binding rates larger or equal $\gamma_{b}>\gamma_{b}^{*}=9.0$, no more spread is possible, since all virus particles get bound to cells immediately.

## 3 Model 2: Long time scale

### 3.1 Basic assumptions and the mathematical model

In this section, we study the spread distances of the viral infection for time scales that include viral replication inside the cells, virion release and cell death ( i.e. more than 16 hours). Specifically, we explain the plaque size results on L929 cell monolayers in [26]. Plaque size refers to the area of dead cells that result from the viral infection. The larger the plaque size, the farther the viral infection has spread. In [26], a monolayer of L929 cells was subjected to infection by reovirus particles. Following a one-hour incubation at $37^{º}C$, a 0.5% agar overlay was introduced onto the cells. Once the agar solidified, the cells were placed back into the $37^{º}C$ incubator for a period of 5 days. Afterward, the cells were treated with 4% paraformaldehyde (PFA) for fixation and the cellular monolayer was stained using a 1% (wt/vol) crystal violet solution. Subsequently, plaque size analysis was performed using the Fiji software with the particle analysis plugin and the results expressed as a relative plaque size to T3wt after normalization T3wt plaque size to 1. A larger plaque size reflects increased efficiencies of one or more steps involved in progressive infection and killing of more and more cells over cycles of virus infection, release, and reinfection.

To analyze the results of plaque size in [26], we assume that the number of cells that can be infected during the experiment is about constant. This is a strong assumption, but we feel that it is justified, since the model shows good results. We include a class of infected cells *I* ( *x* , *t* )  and extend our previous model (1) as


$$\begin{array}{ccc} \begin{array}{ccc} \frac{\partial I}{\partial t} & =\gamma_{b}\nu V-\alpha I & \\ \frac{\partial V}{\partial t} & =D_{\text{V}}\frac{\partial^{2}V}{\partial x^{2}}+\alpha \tilde{b}I-\gamma_{b}V, \end{array} & & \end{array}$$ (8)

where the virus diffusion coefficient $D_{\text{V}}$ and the binding rate $\gamma_{b}$ are the same as before in model (1)-(2). The percentage of binding viruses that lead to infection is denoted by *ν* .  The infected cells die at rate *α* ,  and the burst size of the infectious viruses is denoted by $\tilde{b}.$ Here, we would like to indicate that the rate of virus replication in infected cell are expressed in some papers by parameter *b*, where *b* represents the infected cells death rate  ×  the virus burst size [51,62,77]. This is equivalent in our model to $b=\alpha \tilde{b}$.

In addition to $\gamma_{b}$ and $D_{\text{V}}$, we have three more parameters to estimate: the death rate of infected cells *α*, the percentage of binding virus that lead to an infection *ν*, and the burst size $\tilde{b}$. We estimate these values in the next subsections and summarize the values in Table 3.

**Table 3:** Estimated parameter values of system (8).

Parameter	Description	Value	Unit
$D_{\text{ V}}$	Diffusion coefficient of reovirus	0 . 01 ± 0 . 0015	${\text{mm}}^{2}\;\text{per}\text{ hour}$
$\gamma_{b\text{ T3wt}}$	Binding rate of wild type	0 . 96 ± 0 . 16	per hour
$\gamma_{b\text{ SV5}}$	Binding rate of SV5	0 . 28 ± 0 . 09	per hour
*α*	Death rate of infected cells	0 . 057 ± 0 . 030	per hour
*ν*	Percentage of binding reovirus leads to infection	0.01	cell per virus
$\tilde{b}$	Infectious burst size of reovirus	500-1000	virus per cell
${\tilde{b}}_{\text{ T3wt}}$	Infectious burst size of T3wt	514 ± 114	viable virions per cell
${\tilde{b}}_{\text{ SV5}}$	Infectious burst size of SV5	732 ± 146	viable virions per cell

### 3.2 Death rate of infected cells estimation

**Fig 3:**
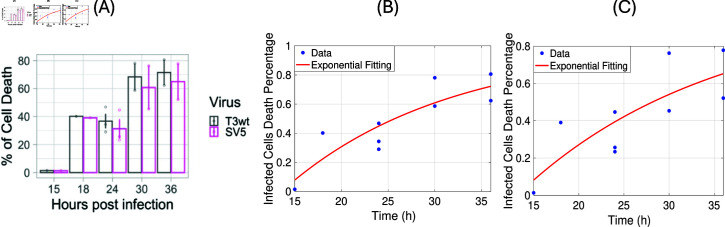
The death rate of infected cells estimation for T3wt and SV5 particles from experiment in Cristi et al. [26]. **(A)**: The percentage of cell death at different time points, picture taken from Fig 6 G in [26]. **(B)+(C)**: Replot of the original cell death data from [26] for T3wt in (B) and SV5 in (C) and our fits $D_{I}(t)=1-e^{-\alpha t}$ as red solid lines.

In [26] the percentage of cell death at time points 15, 18, 24, 30 and 36 hour after inoculation had been measured for each virus type T3wt and SV5. We show these data in Table 4 and illustrate them in Fig 3. While for 15 and 18 hours, only one data point had been measured compared to three data points at time 24 hour and two data points at time 30 and 36 hours each. From the data, we can see that the cells are surviving between 15-24 hours with no significant differences based on the virus type. The death rate of infected cells *α* at different time points can be estimated by fitting the proportion of dead infected cells $D_{I}(t)$ to the exponential function $D_{I}(t)∼1\;-\;e^{-\alpha t}$ using MATLAB, as shown in Fig 3(B) and 3(C). We find no significant difference between the death rate of the T3wt virus and SV5 virus, which is consistent with the data observation. Therefore, we estimate the death rate of infected cells as *α* = 0 . 057 ± 0 . 030 per hour with $R^{2}$ value of 0.76 for T3wt and 0.63 for SV5.

**Table 4:** The percentage of cell death at different time points for T3wt and SV5 particles from the experiment in Cristi et al. [26]. The estimated data range is computed.

Virus Type	Time	*%* of Cell Death	Estimated Data
T3wt	15	1.470	
T3wt	18	40.15	
T3wt	24	34.38	36.72 ± 5.27
T3wt	24	46.79	
T3wt	24	28.99	
T3wt	30	58.64	68.36 ± 9.72
T3wt	30	78.08	
T3wt	36	62.35	71.49 ± 9.14
T3wt	36	80.63	
SV5	15	1.34	
SV5	18	39.05	
SV5	24	25.68	31.25 ± 6.751
SV5	24	44.69	
SV5	24	23.39	
SV5	30	45.34	60.81 ± 15.47
SV5	30	76.28	
SV5	36	52.15	64.99 ± 12.84
SV5	36	77.83	

### 3.3 Viral burst size estimation

**Fig 4:**
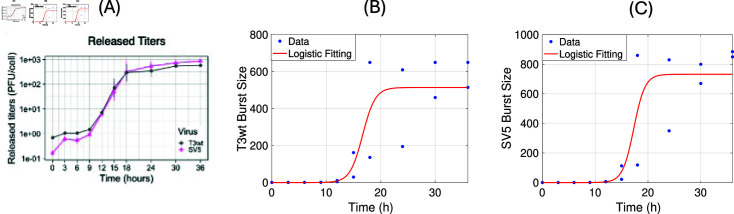
The burst size estimation for T3wt and SV5 particles from experiments in Cristi et al. [26]. **(A)**: The percentage of viral burst size at different time points, taken from Fig 6F in [26]. **(B)+(C)**: Replot of the original burst size data from [26] for T3wt in (B) and SV5 in (C). The logistic fits are shown as red lines.

The burst size of the virus is the number of released new virions from one infected cell. Experimentally in [26], the released number of virions had been measured with two data sets with multiplicity of infection (MOI) 21 for T3wt and 27 for SV5, respectively, at different time points: 0, 3, 6, 9, 12, 15, 18, 24, 30, and 36 hour. The Multiplicity of Infection (MOI) refers to the number of virions that are added per cell during infection. The data are shown in Fig 4. In Fig 4 (B), and (C) we use a logistic fit for these data and estimate the burst size as the carrying capacity value for this logistic fit. We find ${\tilde{b}}_{\text{ T3wt}}=514\pm 114$ viable virions per cell with $R^{2}$ value of 0.79 and ${\tilde{b}}_{\text{SV5}}=732\pm 146$ viable virions per cell with $R^{2}$ value of 0.82, respectively. Note that the confidence intervals for these two values overlap. Hence, as reported already in [26], there is no statistically significant difference in those values. This is an important observation, and we come back to this issue later.

### 3.4 Percentage of infectious viral particles

The results in [78], show that the percentage of bound reovirus that lead to an infection is in the range of $\nu \in [\frac{1}{1000},\frac{1}{100}]$ cells per virus. We choose *ν* = 0 . 01 cell per virus. This means out of 100 binding viruses on average one virus leads to a successful infection.

The parameters values are summarized in Table 3. Now, as all parameters for model (8) are identified, we can begin its analysis.

### 3.5 Viral replication number

A useful quantity for the analysis of our model is the virus replication number (VRN). The VRN can be found by looking at the kinetic part of Eq (8), i.e. by setting diffusion to be zero. Defined in [51] as analogy of the basic reproduction number in epidemiology, the viral replication number denotes the average number of infected cells that result from one infected cell in an otherwise healthy cell population. The viral replication number is used as a measure to quantify the transmissibility in the cell culture. Since the system (8) is linear and the only steady state is  ( *I* , *V* ) = ( 0 , 0 ) ,  then the corresponding eigenvalue problem is


$$\begin{array}{ccc} A\left[\begin{array}{c} I\\ V \end{array}\right]=\lambda \left[\begin{array}{c} I\\ V \end{array}\right]\;\text{ where }\;A=\left[\begin{array}{cc} -\alpha & \gamma_{b}\nu \\ \alpha \tilde{b} & -\gamma_{b} \end{array}\right] & & \end{array}$$ (9)

Condition *det* ⁡  *A* = 0 can be written as $R_{\text{V}}=1,$ where


$$\begin{array}{ccc} R_{\text{V}}=\tilde{b}\nu . & & \end{array}$$ (10)

The stability of the steady state  ( *I* , *V* ) = ( 0 , 0) is determined by $R_{\text{V}}.$ Here, $R_{\text{V}}$ denotes the average burst size. Mathematically, if $R_{\text{V}}<1,$ then both eigenvalues of the matrix A are negative and hence the steady state (0,0) is stable. Biologically, this means that the virus dies out. On the other hand, if $R_{\text{V}}>1,$ then we have one positive eigenvalue and the second eigenvalue is negative. Therefore, the steady state is unstable and hence the virus can spread to infect the neighbouring cells and as a result the virus population grows. From the experimental data, we find that $R_{\text{V}}=\tilde{b}\nu >1,$ where $R_{\text{VT3wt}}=5.14\pm 1.14$ and $R_{\text{VSV5}}=7.32\pm 1.46,$ respectively.

### 3.6 Travelling invasion wave

One way to understand the effect of the binding rate $\gamma_{b}$ on the invasion of the viral infection over the L929 cell culture is an invasion wave analysis. This is a standard method for reaction-diffusion models [79] (like (8)) where an invasion speed is estimated, which describes the speed of the spread of the viral infection over the population of cells. Here we assume that $R_{\text{V}}>1$, such that the virus can grow. Since our model (8) is linear we use the leading edge method, where we focus on the behaviour of the front profile of the invasion, near the steady state  ( *I* , *V* ) = ( 0 , 0 ) .  We use *c* to denote the invasion wave speed and *λ* to denote its exponential decay rate and look for solutions of the form


$$\begin{array}{ccc} \left(I(x,t),V(x,t))=(\varepsilon_{i}e^{-\lambda z},\varepsilon_{v}e^{-\lambda z}\right) & & \end{array}$$ (11)

with *z* = *x* − *ct* and small contants $\varepsilon_{i},\varepsilon_{v}$.

Substituting the Ansatz for  ( *I* ( *x* , *t* ) , *V* ( *x* , *t* ) )  into system (8), we get


$$\begin{array}{cccc} \gamma_{b}\nu \varepsilon_{v}-c\lambda \varepsilon_{i}-\alpha \varepsilon_{i} & =0 & & \\ D_{\text{V}}\lambda^{2}\varepsilon_{v}-c\lambda \varepsilon_{v}+\alpha \tilde{b}\varepsilon_{i}-\gamma_{b}\varepsilon_{v} & =0 & \end{array}$$ (12)

We write system (12) in matrix form *Aε* = 0 , 


$$\begin{array}{ccc} \left[\begin{array}{cc} -(c\lambda +\alpha ) & \gamma_{b}\nu \\ \alpha \tilde{b} & D_{\text{V}}\lambda^{2}-c\lambda -\gamma_{b} \end{array}\right]\left[\begin{array}{c} \varepsilon_{i}\\ \varepsilon_{v} \end{array}\right]=\left[\begin{array}{c} 0\\ 0 \end{array}\right]. & & \end{array}$$ (13)

Thus, to obtain a non-trivial solution, we assume that the determinant of the matrix *A* is zero. The characteristic equation of (13) becomes


$$\begin{array}{ccc} -\left(c\lambda +\alpha \right)\left(D_{\text{V}}\lambda^{2}-c\lambda -\gamma_{b}\right)-\alpha \gamma_{b}\tilde{b}\nu =0. & & \end{array}$$ (14)

Following a method of Volpert [51], we introduce *ϱ* = *cλ* > 0, substitute it into (14) and solve for $c^{2}$ to obtain


$$\begin{array}{ccc} c^{2}=\chi (\varrho ):=\frac{D_{\text{V}}\varrho^{2}(\alpha +\varrho )}{\varrho^{2}+(\alpha +\gamma_{b})\varrho -\alpha \gamma_{b}(\tilde{b}\nu -1)}. & & \end{array}$$ (15)

Next we show that *χ* ( *ϱ* )  has a unique positive minimum at $\varrho^{*}$ such that the minimal wave speed $c^{*}$ is given by


$$\begin{array}{lll} c^{*}=\sqrt{\chi (\varrho^{*})}. & \; & \end{array}$$

Consequently, we find the decay rate of the invasion with minimal speed as


$$\begin{array}{ccc} \lambda^{*}=\frac{\varrho^{*}}{c^{*}}. & & \end{array}$$ (16)

**Fig 5:**
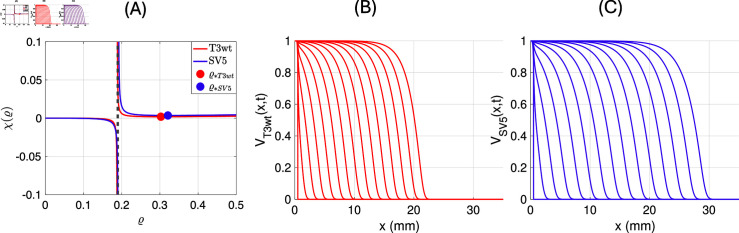
(A) Characteristic function *χ* ( *ρ* )  for the two cases of T3wt (red) and SV5 (purple) and the corresponding minima marked as solid points. (B) and (C) show the travelling wave as function of space, where the wave profiles for different time steps are overlayed. **(B)**: $\gamma_{b\text{T3wt}}=0.96,$ with $\tilde{b}=514,$ and *c* = 0 . 04373 mm per hour. **(C)**: $\gamma_{b\text{SV5}}=0.28,$ with $\tilde{b}=732$ and *c* = 0 . 0599 mm per hour.

For the parameter values that we estimated for the two virus types T3wt and SV5, as reported in Table 3, we plot the curves *χ* ( *ϱ* )  in Fig 5 (A). The function *χ* ( *ϱ* )  has zeros at *ϱ* = 0 ,  and *ϱ* = − *α* ,  which are not relevant since we require *ϱ* > 0 .  Also, *χ* ( *ϱ* )  has a vertical asymptote at


$$\begin{array}{ccc} \varrho_{v}=\frac{-(\alpha +\gamma_{b})+\sqrt{{\left(\alpha +\gamma_{b}\right)}^{2}+4\alpha \gamma_{b}(\tilde{b}\nu -1)}}{2}>0. & & \end{array}$$ (17)

The positive minima, indicated as dots in Fig 5 (A), are right of the asymptote, hence we define


c∗=min ⁡ ϱ>ϱvχ(ϱ). (18)

For the parameter values from Table 3 we get


$$\begin{array}{ccc} c_{\text{T3wt}}^{*}=0.04366\;\text{mm}\;\text{per}\;\text{hour}\;\text{and}\;c_{\text{ SV5}}^{*}=0.05941\;\text{mm}\;\text{per}\;\text{hour}. & & \end{array}$$ (19)

In Fig 5 (B) and 5 (C) we show numerical simulations of the invasion waves for these parameters. We see that the invasion wave of T3wt (B) is slower than the invasion of the SV5 virus (C). If we compute the invasion speeds numerically, we find


$$c_{\text{T3wt}}=0.04373\;\text{mm}\;\text{per}\;\text{hour}\;\text{and}\;c_{\text{SV5}}=0.05990\;\text{mm}\;\text{per}\;\text{hour},$$

which is very close to the theoretical values above (19).

We also determine the corresponding invasion front decay rate ($\lambda^{*}$) by formula (16) and find


$$\begin{array}{ccc} \lambda_{\text{T3wt}}^{*}=6.9\;\text{per}\;\text{mm},\;\text{ and }\;\lambda_{\text{ SV5}}^{*}=5.4\;\text{per}\;\text{mm}. & & \end{array}$$ (20)

### 3.7 Validation of model 2 on invasion front data

In the previous sections we estimated all the model parameters as summarized in Table 3, plus the invasion speeds $c^{*}$ and the decay rates $\lambda^{*}$ at the edge of the invasion front. To validate our model (8) we compare it now to data that have not been used to parameterize the model. The set of data is an experiment in [26] where we use fluorescence measurements of viral load at the edge of the plaques.

**Fig 6:**
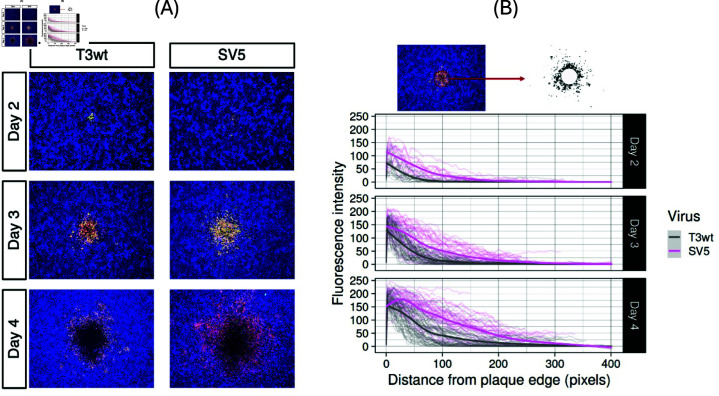
SV5 spread farther than T3wt. **(A)**: Immunofluorescence pictures of T3wt and SV5 plaques formation at days 2, 3 and 4 post infection from Fig 6 H in [26], **(B)**: Quantification of the fluorescence from the edge of the plaque to represent the spread of the virus, where the pink line shows the average value for SV5 and the gray line shows the average value of the wild type T3wt. Taken from Fig 6 I in [26].

When plaques are evaluated by crystal violet staining as in above experiments, then plaque size only reflects the size of clearance produced by killing of cells in the center. Crystal violet staining does not, however, reveal the extent of cells that are infected by virus but are still alive. Therefore, immunofluorescence was used to directly visualize reovirus-infected cells in the margin of the plaques at days 2, 3 and 4 post infection [26] (see Fig 6). In these experiments , SV5 infected cells were found at further distances from the origin than for T3wt. Mathematically, this can be represented by estimating the decay rate *λ* of the invasion front (11) of T3wt and SV5 viruses, which we considered earlier.

**Fig 7:**
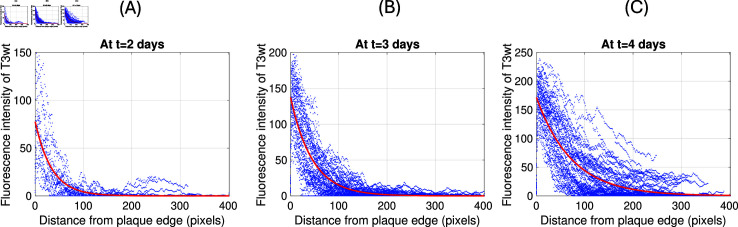
The T3wt invasion front decay rate. We plot in blue the original data from [26] and overlay the exponential decay function in red. **(A)**: *λ* = 0 . 029 at t= 2 days **(B)**:*λ* = 0 . 022 at t=3 days and **(C)**:*λ* = 0 . 014 at t=4 days.

**Fig 8:**
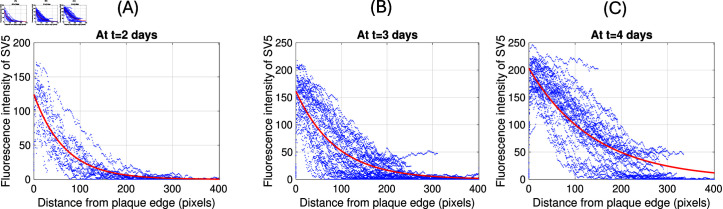
The SV5 invasion front decay rate. We plot in blue the original data from [26] and overlay the exponential decay function in red. **(A)**: *λ* = 0 . 015 at t= 2 days **(B)**: *λ* = 0 . 011 at t=3 days and **(C)**: *λ* = 0 . 007 at t=4 days.

To estimate the decay rates of the T3wt invasion front $\lambda_{\text{T3wt}}$ and the SV5 invasion front $\lambda_{\text{SV5}}$ from the data, we apply MATLAB to fit the data in Fig 6 with an exponential decay function for the value $Fl(x,t)=e^{-\lambda (x-s)},$ where *λ* is the invasion front decay rate and *s* is a shift of the exponential decay function to place it at the best location for the fit. In Figs 7 and 8 we show this fit in red with the corresponding data in blue. In Fig 7 we find the best fit decay rates on days 2, 3, 4 for T3wt to be *λ* = 0 . 029 ± 0 . 001 , 0 . 022 ± 0 . 0004 , 0 . 014 ± 0 . 0003 per pixel, which has a mean value of *λ* = 0 . 022 per pixel. There are 445 pixel per mm, hence we find $\lambda_{\text{T3wt}}=9.8$ per mm. This corresponds well with the previous estimate in (20) of $\lambda_{\text{T3wt}}^{*}=6.9$ per mm. For the supervirus SV5 we find the decay rates *λ* = 0 . 015 ± 0 . 0005 , 0 . 011 ± 0 . 0002 , 0 . 007 ± 0 . 0002 per pixel, with an average of 0.011. This corresponds to $\lambda_{\text{SV5}}=4.9$ per mm. Again, this is very close to the theoretical value of $\lambda_{\text{SV5}}^{*}=5.4$ per mm from (20).

Note that we used 95% confidence intervals for *λ*, which correspond to $\chi_{2}^{2}=5.991$ (with two degrees of freedom). I.e. as parameters vary in the confidence intervals, the log sum of squared errors varies by this value.

### 3.8 The relationship between binding rate and wave speed

The relationship between the binding rate $\gamma_{b}$ and the speed of the viral infection wave over the cell monolayer is very important. To optimize the efficacy of reovirus treatment, we like to find the binding rate $\gamma_{b\text{max}}$ that maximizes the invasion speed $c^{*}$. A formula for $c^{*}$ is given in (18), where the function *χ* ( *ρ* )  and the value of the asymptote $\rho_{v}$ both depend on the binding rate $\gamma_{b}$. We first look at the extreme cases of no binding, $\gamma_{b}=0$, and of immediate binding, $\gamma_{b}\to \infty$.

In the case of no virus binding ($\gamma_{b}=0$), there will be no invasion, since the virus cannot replicate. In this case we expect $c^{*}=0$. Indeed, in case of $\gamma_{b}=0,$ direct calculation in (15) and (17) shows that


$$\varrho_{v}=0\;\text{and}\;\chi (\varrho )=D_{\text{V}}\varrho ,$$

and


c∗=min ⁡ ϱ≥0χ(ϱ)=0.

This means when the binding rate $\gamma_{b}=0,$ the invasion speed for T3wt and SV5 viruses infection is zero.

In the other extreme of immediate binding $\gamma_{b}\to \infty ,$ the virus will bind immediately to cells and will no longer be able to invade any further. So we also expect $c^{*}=0$. In this case we have the singularity of *χ* ( *ρ* )  at $\varrho_{v}=\alpha (\tilde{b}\nu -1)$. For $\rho >\rho_{v}$ we can apply L’Hopital’s rule to *χ* given in (15) to consider the limit as $\gamma_{b}\to \infty$. We find


lim ⁡ γb→∞χ(ϱ)=0,

which implies $c^{*}=0$.

Since in the two extreme cases of $\gamma_{b}$ the invasion speed is zero, and since *χ* and $\rho_{v}$ depend continuously on $\gamma_{b}$ for $\rho >\rho_{v}$, we conclude that there is at least one maximum of $c^{*}$ for some intermediate binding rate $\gamma_{b}\in (0,\infty )$. To find this value we consider the critical points of *χ* ( *ρ* ) :


$$\begin{array}{lll} \begin{array}{ccc} 0= & \chi^{\prime }(\varrho ) & \\ = & \frac{D_{\text{V}}\varrho \left(\varrho^{3}+2(\alpha +\gamma_{b})\varrho^{2}+(\alpha^{2}+4\alpha \gamma_{b}-3\alpha \gamma_{b}\tilde{b}\nu )\varrho -2\alpha^{2}\gamma_{b}(\tilde{b}\nu -1)\right)}{{\left[\varrho^{2}+(\alpha +\gamma_{b})\varrho -\alpha \gamma_{b}(\tilde{b}\nu -1)\right]}^{2}}\\ = & \frac{D_{\text{V}}\varrho P_{3}(\varrho )}{P_{2}^{2}(\varrho )}, \end{array} & \; & \end{array}$$

where


$$P_{3}(\varrho )=\varrho^{3}+2(\alpha +\gamma_{b})\varrho^{2}+(\alpha^{2}+4\alpha \gamma_{b}-3\alpha \gamma_{b}\tilde{b}\nu )\varrho -2\alpha^{2}\gamma_{b}(\tilde{b}\nu -1),$$

and


$$P_{2}(\varrho )=\varrho^{2}+(\alpha +\gamma_{b})\varrho -\alpha \gamma_{b}(\tilde{b}\nu -1).$$

Thus, $\chi^{\prime }(\varrho )=0$ when *ϱ* = 0 or $P_{3}(\varrho )=0.$ Clearly, the coefficient of $\varrho^{3}$ and $\varrho^{2}$ are positive while the sign of the coefficient of $\varrho^{0}$ is negative. The sign of the coefficient of *ϱ* depends on the value of $\gamma_{b}$ after fixing the parameter values of *α* ,  $\tilde{b},$ and *ν* .  Based on Descartes’ rule of signs [80] even if the sign of the coefficient *ϱ* is positive or negative, we have only one positive real root and 2 or zero negative real roots of $P_{3}$. Therefore, there exist a unique $\varrho^{*}>0,$ such that $\chi^{\prime }(\varrho^{*})=0$. Furthermore, at *ϱ* = 0 ,  we have $P_{3}(0)=-2\alpha^{2}\gamma_{b}(\tilde{b}\nu -1)<0,$ with the continuity of $P_{3}(\varrho )$ and being concave up since $P_{3}^{\prime \prime }(\varrho )=6\varrho +4(\alpha +\gamma_{b})>0$ for each *ϱ* ≥ 0 .  Therefore, by intermediate value theorem and mean value theorem, there is only one positive real root i.e $\varrho^{*}>0$ such that $P_{3}(\varrho^{*})=0.$ Thus for each $\gamma_{b}>0,$ $\chi (\varrho^{*})$ is a unique minimum with $P_{3}(\varrho^{*})=0$ for $\varrho^{*}>\rho_{v}.$ The maximum possible invasion speed is then


cmax∗= max ⁡ γb∈(0,∞)c∗(γb).

**Fig 9:**
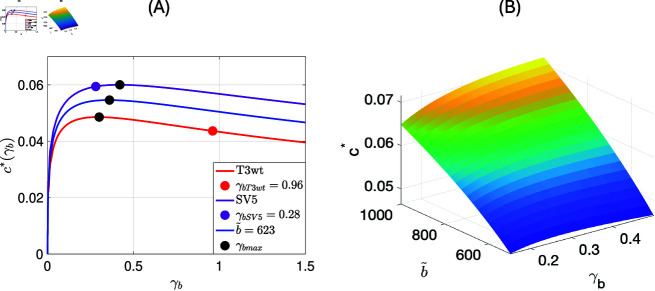
(A) The relationship between the binding rate $\gamma_{b}$ and the invasion speed $c^{*}$ for T3wt, SV5, and when $\tilde{b}=623.$ We plot the function (15) for different values of $\gamma_{b}$ to find the maximum $\gamma_{b\text{max}}$ that leads to maximum wave speed i.e $c_{\text{max}}^{*}.$ The numerical results indicate that for T3wt virus, the $c_{\text{max}}^{*}=0.04858$ at $\gamma_{b\text{max}}=0.29,$ while the $c_{\text{max}}^{*}=0.06002$ at $\gamma_{b\text{max}}=0.42$ for SV5 virus. Finally, when we choose intermediate value of infectious burst size $\tilde{b},$ we find the $c_{\text{max}}^{*}=0.05460$ at $\gamma_{b\text{max}}=0.36$. (B) The values of the minimum wave speed $c^{*}$ when we vary the binding rate $\gamma_{b}$ and the burst size $\tilde{b}.$ The maximum wave speed $c^{*}$ is 0.07154 when $\gamma_{b}=0.5$ and $\tilde{b}=1000.$ The values of the binding rate in (B) have the range $\gamma_{b}\in [0.15,0.5]$.

For the parameter values from Table 3 for T3wt and SV5 we plot the function $c^{*}(\gamma_{b})$ as red line for T3wt and in purple for SV5 in Fig 9 (A). As red and purple points we indicate the estimated binding rates from the data for the corresponding cases, and in black we indicate the maximum of these curves.

- For T3wt (red curve) we observe that the invasion speed could be increased by reducing the binding rate $\gamma_{b}$ from 0.96 per hour to 0.29 per hour. In that case the speed would change from 0.044 mm per hour to 0.048 mm per hour. Expressed in percentage of binding after one hour, we aim to decrease the percentage of binding of T3wt virus from 61.7 % to 25.9 %.
- In the case of SV5 (purple curve) we see that an increase in binding rate from 0.28 to 0.48 would have a small accelerating effect from $c^{*}=0.059$ to 0.060. In other words, we like to increase the percentage of binding of SV5 virus after one hour from 24.4 % to 34.3 %.
- We also notice that this result depends on the burst size of the corresponding virus. As indicated earlier, and also in [26], the difference in the burst sizes is not statistically significant. Hence we add, in blue, the corresponding curve for the mean burst size of $\tilde{b}=62$. The curve is very similar to the red and purple curves and the maximum invasion speed of $c^{*}=0.054$ is found for a binding rate near 0.36.
- We also considered some extreme cases for the burst size of $\tilde{b}=500$ and $\tilde{b}=1000$. For $\tilde{b}=500$ we find $\gamma_{b\text{max}}=0.28$ per hour with corresponding wave speed $c_{\text{max}}^{*}=0.04775\;\text{mm}\;\text{per}\;\text{hour},$ while for the upper bound of burst size $\tilde{b}=1000$ virus per cell we find $\gamma_{b\text{max}}=0.50$ per hour with corresponding wave speed $c_{\text{max}}^{*}=0.07154\;\text{mm}\;\text{per}\;\text{hour}.$

The previous results emphasize the importance of the burst size parameter $\tilde{b}$ in determining the viral spread $c^{*}$ and, as a result, the plaque size. In Fig 9 (B), we determine the minimum wave speed values, denoted as $c^{*}$, by varying the binding rate $\gamma_{b}$ and the burst size $\tilde{b}$. It is observed that for our range of possible burst sizes $\tilde{b}\in [500,1000]$, the maximum wave speed $c^{*}$ reaches 0.07154 when $\gamma_{b}=0.5$ and $\tilde{b}=1000$.

We observe that the ratio $\frac{\gamma_{\text{max}}}{\tilde{b}}$ in Table 5 and Table 6 remains nearly constant for each maximum binding rate $\gamma_{\text{max}}$ corresponding to the burst size $\tilde{b}$. Hence, we might use the ratio $\frac{\gamma_{\text{max}}}{\tilde{b}}$ as a benchmark to assess how closely the experimental results approach the maximum required viral spread speed. The average number of ratio $\frac{\gamma_{\text{max}}}{\tilde{b}}$ is 0.00056. Therefore, the optimal binding rate and burst size have a ratio of about 0.00056.

**Table 5:** The infectious burst size, the binding rate, *%* of binding viruses and the corresponding wave speed for the wild type T3wt, intermediate infectious burst size, and SV5 virus.

	$\gamma_{b}$	*%* of Binding	$c^{*}$	$\tilde{b}$	$\frac{\gamma_{\text{ max}}}{\tilde{b}}$
T3wt Data	0.96	61.7	0.04366	514	
Max T3wt	0.29	25.2	0.04858	514	0.00056
Intermediate Max	0.36	30.2	0.05460	623	0.00058
SV5 Data	0.28	24.4	0.05941	732	
Max SV5	0.42	34.3	0.06002	732	0.00057

**Table 6:** The ratio of infectious burst size, the corresponding maximum binding rate, the maximum wave speed and the corresponding plaque size. The plaque size is computed with Model 3 as described in Sect 4.

$\tilde{b}$	$\gamma_{\text{ bmax}}$	$\frac{\gamma_{\text{ bmax}}}{\tilde{b}}$	$c_{\text{ max}}^{*}$	Plaque Size
virus per cell	per hour	cell per (virus × hour)	mm per hour	${\text{mm}}^{2}$
500	0.28	0.00056	0.04775	5
550	0.32	0.00058	0.05065	20
600	0.36	0.00060	0.05338	32
650	0.36	0.00055	0.05600	43
700	0.39	0.00056	0.05848	55
750	0.43	0.00057	0.06090	69
800	0.47	0.00059	0.06316	82
850	0.47	0.00055	0.06538	95
900	0.51	0.00057	0.06753	106
950	0.51	0.00054	0.06959	121
1000	0.50	0.00050	0.07154	129

## 4 Model 3: Plaque size

### 4.1 Plaque size experiments

In [26] an experiment is designed to measure the plaque size of the T3wt and SV5 viruses. They reported the relative areas $A_{\text{SV5}}∕A_{\text{T3wt}}$ and found that the relative value of plaque size between SV5 and T3wt after 5 days varies between 3.4530 to 5.1248. This means that after 5 days, the plaque size of SV5 virus is about 4 times larger than the plaque size of the T3wt virus. We would like to point out the reason to measure the relative value of plaque size. It was observed that repeat experiments lead to different plaque sizes, due to variables that are out of control of the experimentalist such as cell viability, humidity, person performing the experiments, etc.. However, the relative plaque size difference of a factor of 4 were similar in all experiments. The reported values for $A_{\text{SV5}}∕A_{\text{T3wt}}$ are


7.5575,4.3643,3.5976,3.3551,4.2194,4.0628,4.256,3.7092,3.4748.

with mean and standard error


$$\frac{A_{\text{SV5}}}{A_{\text{T3wt}}}=4.2889\pm 0.8359,$$

which we like to confirm with our model.

To properly keep track of the plaque sizes, we now include the cancer cell compartment *C* ( *x* , *t* )  explicitly and we solve the model on a 2-dimensional square domain. The plaques correspond to regions of dead cancer cells, and in our modelling we identify those as regions where *C* ( *x* , *t* )  is below a small threshold. Our previous model (8) is now extended to **Model 3:**


$$\begin{array}{ccc} \begin{array}{ccc} \frac{\partial C}{\partial t} & =-\gamma_{b}\nu CV, & \\ \frac{\partial I}{\partial t} & =\gamma_{b}\nu CV-\alpha I,\\ \frac{\partial V}{\partial t} & =D_{\text{V}}\Delta V+\alpha \tilde{b}I-\gamma_{b}V, \end{array} & & \end{array}$$ (21)

with homogeneous Neumann boundary conditions on a square domain of size 30 × 30. Here we use the same parameters as identified earlier, see Table 3.

**Fig 10:**
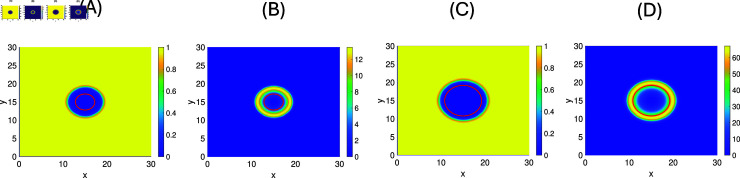
The plaque sizes at 5 days. **(A)+(B)**: T3wt spread with cancer cell density in (A) and viral concentration in (B). The *C*-level of 1*%* is indicated as a red line. **(C)+(D)**: SV5 infection with cancer cells in (C) and SV5 in (D). The computed plaque sizes are indicated in the red circle in (A) which is 15.2053 for T3wt virus and 55.4177 for SV5 virus in (C). Therefore, the relative plaque size of SV5 related to T3wt is 3.6446.

We numerically solve our model (21) with virus inoculated in the center of a two-dimensional domain (see Fig 10). We estimate the plaque sizes after 5 days with threshold for cancer cells of 1%, indicated as a red line in the figures. We find a ratio $\frac{A_{\text{SV5}}}{A_{\text{T3wt}}}=\frac{55.4177}{15.2053}\approx 3.6,$ which is very close to the experimental ratio mentioned above. We note that the T3wt and SV5 invasion forms a hollow ring spread pattern (see Fig 10). Such invasion patterns are typical for virus infections of tissues, and were also previously found in [67,69].

**Fig 11:**
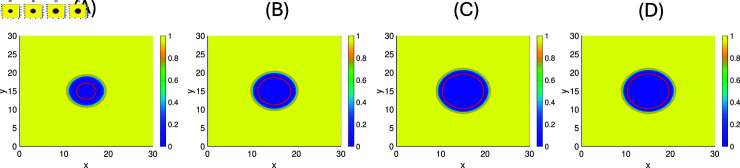
The plaque sizes of T3wt at t=5 days when $\gamma_{b}=0.96$ with different burst sizes $\tilde{b}$. **(A)**: $\tilde{b}=514$ **(B)**: $\tilde{b}=623$ **(C)**: $\tilde{b}=732$ and **(D)**: $\tilde{b}=1000.$ The threshold= 1 %.

**Fig 12:**
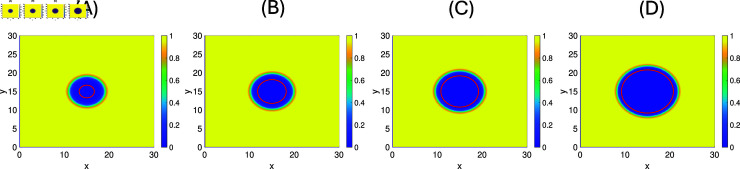
The plaque sizes of SV5 at t=5 days when $\gamma_{b}=0.28$ with different burst size $\tilde{b}$. **(A)**: $\tilde{b}=514$ **(B)**: $\tilde{b}=623$ **(C)**: $\tilde{b}=732$ and **(D)**: $\tilde{b}=1000.$ The threshold=1 %.

Furthermore, we perform simulations of this model for a few chosen parameter values to see the dependence on $\gamma_{b}$ and $\tilde{b}$. In Figs 11 and 12, we fix all parameters as in Table 3, while the burst size $\tilde{b}$ is varied as follows: 514 (T3wt), 623 (average), 732 (SV5), and 1000 (max). A notable increase in plaque size is observed for increased burst size.

The paper [26] presents an additional dataset that we have not included here. These data pertain to plaque experiments conducted under the administration of the drug neuraminidase. Neuraminidase, known as a cancer chemotherapy agent, reduces the binding affinity of the virus. Our extended model (21) appears to offer the appropriate level of detail to simulate these experiments, and we are currently engaged in discussions regarding the specifics of this modeling.

## 5 Conclusion

This study employs a reaction-diffusion model to investigate key aspects of viral infection dynamics of cancer cell monolayers. Specifically, we explore the impact of the binding rate on the spread of the viral infection over the monolayer, the correlation between viral invasion speed and binding rate, and the repercussions of reducing binding rate on plaque size. Two distinct time scales are considered: a short duration (less than 16 hours) focusing on viral spread preceding cell death and replication events, and a longer time scale addressing viral infection between cells. All the parameters in our models are estimated using data from [26].

Our model establishes that the maximum speed of viral infection aligns with a fine balance of viral binding and burst size. The binding has to be fast enough to allow for efficient cell infection, but in the absence of fast flowing medium, it also has to be weak enough to allow the virus to spread longer distances before binding. These observations from the mathematical modelling contradict the common belief that increased binding rate leads to increased viral infection. If binding is increased too much, the viral particles get trapped locally and cannot invade into the remaining tissue. For the specific case of reovirus considered here, our findings indicate that 25.9% binding after one hour is optimal for T3wt virus to achieve the maximum viral spread rate, whereas the SV5 virus infection is optimized for approximately 34.3% binding after one hour. Based on these findings, reovirus mutant can now be screened for binding efficiency with a specific objective to find a mutant that can best fulfil this postulated optimum. In previous studies, reovirus plaque size on cancer cells in vitro correlated with significantly improved oncolytic activity in vivo. We can predict therefore that a reovirus mutant of burst size $\tilde{b}=1000$ with 39.3 % binding would not only generate larger plaques than T3wt on cancer cells in vitro, but reduce tumor size and improve survival also relative to T3wt in mouse models of breast cancer.

While reduced cell attachment has been previously associated with altered pathogenesis in some viruses [42–49], our mathematical analysis, coupled with the empirical data presented in our companion paper [26], provides the first evidence that decreased cell attachment can enhance viral oncolytic activity. Focusing on reovirus oncolytic potential, our findings reveal that the optimal level of cell attachment is context-dependent. In natural reovirus infections of the enteric tract, rapid and strong cell attachment is crucial for establishing infection in a dynamic environment. Conversely, in the relatively static tumor microenvironment, strong attachment likely limits the spread of progeny virus particles, confining the infection to localized areas. We propose that other oncolytic viruses could similarly benefit from optimized cell attachment properties. Specifically, reduced cell binding could promote wider dissemination within tumors, potentially leading to more extensive direct tumor cell killing and enhanced anti-tumor immunity. This insight opens new avenues for engineering oncolytic viruses with tailored attachment properties to maximize their therapeutic efficacy.
